# Mesenchymal stem cell-derived extracellular vesicles, osteoimmunology and orthopedic diseases

**DOI:** 10.7717/peerj.14677

**Published:** 2023-01-24

**Authors:** Maoxiao Ma, Guofeng Cui, Youwen Liu, Yanfeng Tang, Xiaoshuai Lu, Chen Yue, Xue Zhang

**Affiliations:** 1 Department of Orthopedics, Luoyang Orthopedic Hospital of Henan Province, Orthopedic Hospital of Henan Province, Luoyang, Henan, China; 2 Department of Orthopedics, Luoyang Central Hospital Affiliated to Zhengzhou University, Luoyang, Henan, China

**Keywords:** Mesenchymal stem cell, Extracellular vesicles, Osteoimmunology, Orthopedic diseases

## Abstract

Mesenchymal stem cells (MSCs) play an important role in tissue healing and regenerative medicine due to their self-renewal and multi-directional differentiation properties. MSCs exert their therapeutic effects mainly via the paracrine pathway, which involves the secretion of extracellular vesicles (EVs). EVs have a high drug loading capacity and can transport various molecules, such as proteins, nucleic acids, and lipids, that can modify the course of diverse diseases. Due to their ability to maintain the therapeutic effects of their parent cells, MSC-derived EVs have emerged as a promising, safe cell-free treatment approach for tissue regeneration. With advances in inflammation research and emergence of the field of osteoimmunology, evidence has accumulated pointing to the role of inflammatory and osteoimmunological processes in the occurrence and progression of orthopedic diseases. Several studies have shown that MSC-derived EVs participate in bone regeneration and the pathophysiology of orthopedic diseases by regulating the inflammatory environment, enhancing angiogenesis, and promoting the differentiation and proliferation of osteoblasts and osteoclasts. In this review, we summarize recent advances in the application and functions of MSC-derived EVs as potential therapies against orthopedic diseases, including osteoarthritis, intervertebral disc degeneration, osteoporosis and osteonecrosis.

## Introduction

As early as the 1970s, some studies showed that certain immune cells secrete osteoclast-activating factor, which opened new lines of investigation at the interface of bone biology and immunology (Horton et al., 1972), ultimately leading to a field that became known as “osteoimmunology” at the beginning of this century (Takayanagi et al., 2000). Work in this field has established that T cells, B cells and macrophage-related immune cells (Ono & Takayanagi, 2017) interact with the bone marrow directly, as well as indirectly through transcription factors, cytokines and their receptors (Fierro, Nolta & Adamopoulos, 2017).

Mesenchymal stem cells (MSCs) have a strong capacity for self-renewal and multi-directional differentiation, making them useful for tissue healing and regenerative medicine (Ding, Shyu & Lin, 2011). MSCs can differentiate into mesodermal tissues, such as osteoblasts, chondrocytes and adipocytes; and they can regulate immune responses (Pittenger et al., 1999). These characteristics have made MSCs an important focus in osteoimmune research. MSCs play a key role in bone formation. They differentiate into osteoblasts by expressing transcription factors Runx2 and osterix (Tjempakasari, Suroto & Santoso, 2021). They can also secrete alkaline phosphatase to synthesize extracellular matrix such as type I collagen, osteopontin (OPN), osteocalcin (OCN), further mineralizing the matrix to form bone tissue (Birmingham et al., 2012; Park et al., 2021). At the same time, MSCs regulate innate immunity in various ways. They can inhibit the differentiation and maturation of neutrophils and monocytes, or promote the polarization of macrophages by secreting different cytokines, which can promote tissue healing (Chen et al., 2008; Cho et al., 2014; Jiang et al., 2016; Zhang et al., 2010; Zhang et al., 2004). MSCs participate in adaptive immune regulation by inhibiting T cell proliferation, regulating B cell proliferation and differentiation, inhibiting B cell apoptosis, and inducing regulatory B cells (Bregs) (Carreras-Planella et al., 2019; Tian et al., 2022).

MSCs perform their functions *via* paracrine mechanisms partially mediated by extracellular vesicles (EVs) (Mendt, Rezvani & Shpall, 2019). EVs are lipid bilayer-enclosed compartments secreted by various cell types (Ibrahim & Khan, 2022). EVs contain a wide range of nucleic acids, proteins, and lipids, which can exert several functions through diverse mechanisms and pathways (Ibrahim & Khan, 2022). Because of their key role in pathophysiology, EVs have become a new strategy for diseases affecting the cardiovascular system, kidney, liver, lung, and nervous system (Gatti et al., 2011; Lai et al., 2022; Zhao et al., 2020a). MSC-derived EVs are also widely used in orthopedics. MSCs-derived EVs can mediate the formation of bone or cartilage by regulating the differentiation of osteoblasts, osteoclasts and chondrocytes, as well as by regulating osteoimmune processes, thereby influencing the course of orthopedic diseases (Luo et al., 2021; Meng & Qiu, 2020; Tsiapalis & O’Driscoll, 2020; Wei et al., 2019; Zhao et al., 2020a; Zhao et al., 2020b).

In recent years, EVs has been found to play a variety of roles in the process of disease, but it has not been described in the field of osteoimmunity in orthopedic diseases. In light of recent advances in osteoimmunity, this review surveys recent developments in our understanding of MSC-derived EVs in orthopedic diseases. First, we focus on the immunomodulatory effects of MSCs, then we discuss the types and biological characteristics of EVs to clarify their therapeutic advantages. Finally, we describe progress in elucidating how MSC-derived EVs mediate bone immunity in orthopedic diseases. Understanding the various characteristics and functions of MSC-derived EVs provides new insights into the pathophysiology of orthopedic diseases, which may help develop more effective treatments.

### Osteoimmunology: a combination of bone biology and immunology

Highlighting the interaction between skeletal system and immune system, osteoimmunology aims to explore the relationship between bone biology and immunology. The detection of activated T lymphocytes to express receptor activator for nuclear factor *κ*B ligand (RANKL) is the most direct evidence of the interaction between the skeletal system and the immune system (Yao et al., 2021). In bone biology, activation of T cells during inflammatory conditions leads to enhanced production of RANKL and tumor necrosis factor-α (TNF-α), thereby promoting osteoclastogenesis and subsequent bone loss in various inflammatory and autoimmune conditions (Colucci et al., 2004; Dar et al., 2018). While immune cells such as macrophages and neutrophils are important for resolving inflammation and promoting repair, they can also contribute to orthopedic diseases (Castanheira & Kubes, 2019; Jamalpoor et al., 2018; Saxena, Routh & Mukhopadhaya, 2021). With an increasing amount of evidence linking the impact of abnormal immunoregulation to bone biology, the dysfunction of the immune system has been considered as an indispensable role to the occurrence and progression of orthopedic diseases (Goodman & Maruyama, 2020; Lee et al., 2019; Zhou et al., 2022). Our understanding of this dysfunction has advanced rapidly with the combination of bone biology and immunology—long considered separately from each other—into the field of osteoimmunology (Ma et al., 2022). This field has developed into a research hotspot as well as an important research direction for orthopedic diseases research.

### Immunoregulation mediated by MSCs

MSCs can regulate innate immunity in different ways. Upon infection with microorganisms, MSCs cause the accumulation of neutrophils in the body by secreting macrophage migration inhibitor factor (MIF), thus inducing the body to eliminate the foreign invaders (Brandau et al., 2010). At the same time, in order to minimize tissue damage, MSCs prevent excessive neutrophil activity by producing superoxide dismutase 3 (SOD3), reducing the level of superoxide anion, and inhibiting the formation of neutrophil extracellular trap (NET) and the release of tissue damage protease (Jiang et al., 2016). MSCs also secrete chemokines such as Chemokine Ligand 2 (CCL2), CCL3 and CCL12 to promote the migration of monocyte macrophages to injured tissue, thus promoting tissue healing (Chen et al., 2008). MSCs downregulate CD40, CD80, CD86 and HLA-DR, inhibiting monocyte differentiation and maturation, restricting cytokine production by dendritic cells and activation of T cells (Zhang et al., 2004). In addition, MSCs can promote the polarization of macrophages to the anti-inflammatory phenotype (M2) (Zhang et al., 2010). For example, co-culturing macrophages with MSCs polarized the macrophages to M2 by upregulating arginase 1 and CD206 (Cho et al., 2014; Zhang et al., 2010). This increased the secretion of interleukin-4 (IL-4) and IL-10 while reducing the production of cell chemoattractant protein 1(MCP-1), TNF-α, IL-1β and inducible nitric oxide synthase (iNOS).

MSCs also regulate the adaptive immune system. They effectively inhibit the proliferation of T cells, such as in animal models of graft-versus-host disease (Bartholomew et al., 2002). The inhibitory effect of MSCs on T cell proliferation is thought to be caused by the release of transforming growth factor-β (TGF- *β*) and hepatocyte growth factor (HGF), as well as the decrease of cyclin D2 and the increase of p27^kip1^ expression in T cells, resulting in the inhibition of T cell proliferation (Nicola et al., 2002; Glennie et al., 2005). MSCs also regulate B cell proliferation and differentiation and inhibit B cell apoptosis, thereby dampening adaptive immune responses. MSCs induce and regulate Bregs, in particular by promoting the secretion of IL-10, ultimately promoting B cell proliferation and differentiation and suppressing immune responses (Corcione et al., 2006).

### Types and biological characteristics of EVs

EVs mediate communication between cells and promote osteogenesis, bone regeneration and mineralization, as well as formation of vascular networks (Zhao et al., 2020a). MSCs-derived EVs present several advantages over the corresponding cell-based therapies: lower cytotoxicity, immunogenicity low enough to allow allogeneic transplantation, lower risk of iatrogenic tumor formation, more convenient manufacture and storage, greater stability, and longer-lasting biological activity (Barile et al., 2014; Keshtkar, Azarpira & Ghahremani, 2018; Mendt, Rezvani & Shpall, 2019; Tsiapalis & O’Driscoll, 2020). Below we present the different types of EVs and describe their biological characteristics and activities.

#### Exosomes

Exosomes are cell-derived vesicles that are present in many and perhaps all biological fluids. Their diameter is between 40 and 200 nm, and their density ranges between 1.13 to 1.19 g/ml (Hessvik & Llorente, 2018; Pol et al., 2012). They form through endocytosis of the plasma membrane, then the inner membrane sprouts inward to form multivesicular bodies. These bodies later fuse with the plasma membrane to secrete internal vesicles (Hessvik & Llorente, 2018; Pol et al., 2012). As a result, exosomes are vesicular, membrane-rich cup-shaped structures with a complex composition of protein, nucleic acids, lipids and other metabolites (Hessvik & Llorente, 2018; Mendt, Rezvani & Shpall, 2019; Pol et al., 2012). Exosomes serve as transport vehicles, playing an important role in intercellular communication (Pol et al., 2012; Zhao et al., 2020a). Exosomes contain proteins involved in membrane transport and fusion, such as Rab, annexins, and flotillin, as well as components of the endosomal sorting complex required for transport, such as Alix, tumor susceptibility gene 101, heat shock protein 70, integrins, and tetraspanin molecules CD9, CD63, CD81, CD82 and HSP70 (Kordelas et al., 2014; Pol et al., 2012).

#### Microvesicles

Microvesicles are present in most biological fluids, their diameter ranges between 200 and 2,000 nm, and their density ranges between 1.16 and 1.19 g/ml (Keshtkar, Azarpira & Ghahremani, 2018; Pol et al., 2012; Vig & Fernandes, 2022; Zhao et al., 2020a). In contrast to exosomes, microvesicles form directly through protrusion and budding of the cell membrane, and they can alter the behavior of target cells by transporting intracellular proteins (Pol et al., 2012). The size ranges of microvesicle and exosomes may overlap, which is important to remember when EVs are isolated from body fluids. Microvesicles contain CD40, matrix metalloproteases (MMP), caspases, selectin, integrins and cytoskeletal protein, and their cell membrane is highly rich in cholesterol, phosphatidylserine and diacylglycerol (Lai, Lichty & Bowdish, 2015; Ratajczak & Ratajczak, 2020).

#### Apoptotic bodies

When cells undergo apoptosis, they release caspase-3 and rho-related kinase I, then form vesicles called apoptotic bodies or apoptotic vesicles (Ela et al., 2013; Todorova et al., 2017). These bodies produce anti-inflammatory or tolerogenic reactions when absorbed by adjacent cells (Pol et al., 2012). Apoptotic bodies are membrane vesicles that form through lysis or autophagy after apoptosis, they have a diameter of 500–5,000 nm, and their density ranges between 1.16 and 1.28 g/ml (Pol et al., 2012). Specific surface markers of apoptotic bodies include DNA, tumor antigens, phosphatidylserine and histones (Bergsmedh et al., 2001). Inappropriate clearance of apoptotic vesicles is considered to be the primary cause of systemic autoimmune disease (Pol et al., 2012).

### Immunomodulatory functions of MSCs-derived EVs in orthopedic diseases

Their immunosuppressive and anti-inflammatory properties make MSCs promising for many therapeutic applications (Griffin et al., 2013). Acting as a bridge between MSCs and recipient cells, MSCs-derived EVs carry a variety of nucleic acid, protein and other bio-active molecules to play anti-inflammatory and immunomodulatory roles in a variety of tissues and organs (Phinney & Pittenger, 2017). This type of EVs can (i) accelerate bone formation and inhibit bone resorption by regulating the differentiation of osteoblasts and osteoclasts through the promotion of early osteogenic markers expression, such as alkaline phosphatase and bone morphogenetic protein 2; (ii) enhance the regeneration of damaged cartilage by inducing proliferation, migration, and matrix synthesis of chondrocytes; (iii) enhance chondroprotection through reducing pro-inflammatory mediators production and increasing anti-inflammatory cytokine production; and (iv) inhibit inflammatory responses through promoting the polarization of macrophage towards to the M2 phenotype, decrease the secretion of the pro-inflammatory cytokines of TNF-α, IL-1, and IL-6, and augment the production of the anti-inflammatory cytokine of IL-10, thereby influencing the development of many orthopedic diseases (Luo et al., 2021; Meng & Qiu, 2020; Tsiapalis & O’Driscoll, 2020; Wei et al., 2019; Zhao et al., 2020a; Zhao et al., 2020b).

#### Osteoarthritis

Osteoarthritis is an age-related degenerative joint disorder that affects ∼7% of the global population (Hunter, March & Chew, 2020), and it is characterized by articular cartilage destruction, synovial inflammation, sclerosis of subchondral bone, and loss of extracellular matrix (ECM) (Sacitharan, 2019; Taghiyar et al., 2021). The dysfunction of osteoimmunology has been confirmed to be closely related to the occurrence and and progression of osteoarthritis. In the early stage of osteoarthritis, macrophages and neutrophils infiltrate in the synovial and produce inflammatory factors such as IL-1 and TNF-α (Woodell-May & Sommerfeld, 2020). These inflammatory cytokines stimulate chondrocytes to produce matrix degrading enzymes, which can increase matrix degradation and accelerate the progress of osteoarthritis (Griffin & Scanzello, 2019; Woodell-May & Sommerfeld, 2020). In recent years, MSC-derived EVs have emerged as a promising approach to treating osteoarthritis for its immunoregulatory functions (Sokolove & Lepus, 2013). Several studies have shown that EVs can alleviate the development of osteoarthritis by inhibiting inflammation, protecting cartilage and regulating extracellular matrix (ECM) synthesis and catabolism (Kim et al., 2021; Mianehsaz et al., 2019). For instance, exosomes derived from bone marrow mesenchymal stem cells (BMSCs) have been found to alleviate cartilage damage, reduce osteophyte formation and synovial macrophage infiltration, inhibit the production of activated pro-inflammatory phenotype (M1) macrophages, and promote the generation of activated M2 macrophages (Zhang et al., 2020a). Another study found that EVs derived from human umbilical cord mesenchymal stem cells (hUCMSCs), by carrying proteins and miRNAs, produced anti-inflammatory and immunomodulatory effects, thus interfering with the occurrence and development of osteoarthritis (Li et al., 2022). EVs can also act through PI3K-Akt signaling to promote polarization of M2 macrophage and reduce levels of pro-inflammatory factors TNF-α, IL-1 and IL-6, giving them strong immunomodulatory potential (Li et al., 2022). In another study, hUCMSC-derived EVs polarized macrophages to the M2 type, based on analysis of the polarization markers CD14, IL-1β, IL-10 and CD206 (Tang et al., 2021). These EVs through PI3K-Akt signaling to stimulate chondrocyte activity and matrix remodeling within the inflammatory environment. MSCs-derived EVs have been shown to contain miRNAs that have been associated with development and progression of osteoarthritis, and some miRNAs from EVs derived from adipose mesenchymal stem cells (ASCs) have been shown to exert anti-inflammatory and protective effects on macrophages, T cells and inflamed chondrocytes *in vitro* (Ragni et al., 2021). For example, miR-155-5p is crucial for regulatory T cell (Treg) proliferation because it induces the IL-2 receptor; while miR-24-3p overexpression significantly inhibits macrophage activation and M1 polarization (Ragni et al., 2021).

Protecting cartilage is one of the important therapeutic aims in osteoarthritis. BMSCs-derived EVs can promote the proliferation and migration of chondrocytes, while reducing apoptosis by downregulating IL-1β-activated pro-inflammatory signal involving Erk1/2, PI3K-Akt, TAK1 and NF- *κ*B. This alleviates osteoarthritic cartilage injury *in vitro* (Li et al., 2020). In addition, ASCs-derived EVs can promote the proliferation and migration of chondrocytes and slow the development of osteoarthritis by inhibiting IL-1β and inflammatory responses (Woo et al., 2020).

Taken together, the available evidence indicates that MSC-derived EVs can alleviate the symptoms of osteoarthritis by preventing the apoptosis of chondrocytes while promoting their proliferation and migration, by regulating immune cells and by inhibiting inflammatory responses. Nevertheless, their complex composition and multiple functions need to be further explored.

#### Osteoporosis

Osteoporosis is a chronic metabolic bone disease that arises through an imbalance between osteogenesis and osteoclastogenesis (Armas & Recker, 2012). With the development of society and longer average life expectancy, this disease has become one of the most widespread and complex skeletal disorders worldwide, especially among postmenopausal women and the elderly (Clynes et al., 2020), yet effective treatments are lacking (Dimitriou et al., 2011; Ensrud & Crandall, 2019). An increasing number of evidence has attributed the pathogenesis of osteoporosis to the dysfunction of immunoregulation (Livshits & Kalinkovich, 2022). Immune cells such as over-activated M1 macrophages, neutrophils, and mast cells release a great quantity of reactive oxygen species (ROS), pro-inflammatory cytokines or chemokines (Dou et al., 2018; Livshits & Kalinkovich, 2022). These inflammatory mediators cause bone loss and subsequent osteoporosis by directly or indirectly inhibiting osteogenic differentiation of BMSCs and inducing apoptosis of osteocytes, osteoblasts and BMSCs.

EVs secreted by MSCs exert important immunoregulatory effects on bone repair in osteoporosis. For example, ASCs-derived EVs can significantly inhibit the osteoclast differentiation of macrophages, promoting the migration of BMSCs (Lee et al., 2021). Those EVs inhibited osteoclast differentiation through osteoprotegerin (OPG), mir-21-5p and let-7b-5p, and they downregulated genes related to bone resorption. In addition, the lncRNA NRON inside BMSCs-derived EVs that have been induced by bioactive glass nanoparticles can activate transcription factors of NFAT family, inhibiting the nuclear translocation of nuclear factor of activated T-cells cytoplasmic 1 (NFATc1) and thereby the osteoclast differentiation of macrophages (Yang et al., 2022). Another study showed that hUCMSCs-derived exosomes can effectively inhibit the differentiation of macrophages into osteoclasts, enhance bone formation, reduce bone marrow fat accumulation and reduce bone resorption in osteoporotic mice, ultimately reducing bone loss (Hu et al., 2020). ASCs-derived exosomes effectively inhibited osteocyte apoptosis induced by hypoxia and serum deprivation, and they exerted these effects by upregulating Bcl-2/Bax and by suppressing the production of reactive oxygen species, production of cytochrome C, activation of caspases-3/-9 and downregulation of the expression of RANKL (Ren et al., 2019). Tissue engineering has developed rapidly, and MSCs-derived exosomes have shown strong potential in this regard (Shahrezaee et al., 2018; Wang et al., 2020). In one study, MSCs-derived exosomes were modified with polycaprolactone (PCL) and *S*-nitrosoglutathione (GSNO), and the resulting vesicles significantly reduced the inflammation stimulated by inflammatory macrophages and inflammatory cytokines (IL-6, TNF-α, iNOS, IL-1β). The modification also further accelerated osteogenic differentiation of MSCs (Wang et al., 2020).

The inflammatory response plays a crucial role in bone formation, during which the immune system responds to a variety of cytokines to recruit and activate a variety of cell types, such as MSCs (Kovach et al., 2015). MSCs-derived EVs play an important role in regulating inflammation, so whether MSCs-derived EVs can interfere with the pathogenesis of osteoporosis by regulating inflammation is an important direction for future research.

These results indicate that MSCs-derived EVs may serve as a promising agent for osteoporosis treatment by regulating the differentiation of osteoblasts and osteoclasts and promoting bone regeneration. As the exploitation of EVs expands, studying the various reactions that they mediate will be a direction for future study.

#### Intervertebral disc degeneration

Intervertebral disc degeneration (IDD) is a pathological condition associated with degeneration of the intervertebral disc, which comprises an inner nucleus pulposus surrounded by an annulus fibrosus (Dowdell et al., 2017). IDD progression is characterized mainly by increased cell death, ECM destruction (Freemont et al., 2002), and accumulation of inflammatory factors (Ding, Shao & Xiong, 2013). Although MSCs themselves were once considered a potential treatment against IDD due to their strong ability to differentiate and modulate immune responses, recent research suggests that MSCs-derived EVs and their miRNAs may exert even stronger therapeutic effects (Lu et al., 2021).

Inflammatory reactions are one of the important pathological drivers of IDD. During development of the disease, IL-1β in the nucleus pulposus significantly increases, followed by an increase in the levels of inflammatory mediators such as COX-2, NO, and NOS (Zhu et al., 2020). MSCs-derived exosomes can interfere with the occurrence and development of IDD by inhibiting apoptosis of nucleus pulposus cells (NPCs); reversing IL-1β-induced secretion of the inflammatory cytokines TNF-α, IL-6, IL-8, IL-12 and IL-18; and activating mitogen-activated protein kinase (MAPK) (Zhu et al., 2020). In a rat model of IDD, EVs secreted by metformin-treated MSCs ameliorated intervertebral disc cell senescence (Cui & Zhang, 2021), and the miR-129-5p within the EVs inhibited apoptosis of nucleus pulposus cells, ECM degradation, and polarization of M1 macrophages (Cui & Zhang, 2021). In addition, BMSC-derived exosomes inhibited activation of the NACHT, LRR, and PYD domain-containing protein 3 (NLRP3) inflammasome in NPCs, producing anti-inflammatory effects (Xia et al., 2019). BMSC-derived exosomes can downregulate levels of ROS in NPCs and thereby attenuate their apoptosis, while also downregulating ECM-degrading proteases to protect the ECM; both effects can protect against IDD (Xia et al., 2019). Overproduction of reactive oxygen species is common in degenerative IDD: the oxidative stress enhances matrix degradation and inflammation and reduces the number of viable, functional cells in the IDD microenvironment (Hu et al., 2022). MSC-derived exosomes carrying miR-31-5p act *via* the ATF6-related ER-stress pathway to inhibit apoptosis and calcification in endplate chondrocytes (EPCs) under oxidative stress (Xie et al., 2020).

These studies have shown that MSC-derived EVs and their miRNAs can significantly mitigate IDD through regulation of inflammatory responses. Additional research should focus on how MSC-derived EVs can modulate inflammatory responses by immune cells as a strategy to delay or even reverse IDD.

#### Osteonecrosis

Osteonecrosis, also known as ischemic necrosis, is a multifactorial orthopedic disease that is progressive, devastating and refractory (Hernigou et al., 2016). It is characterized by a stereotypical pattern of cell death and a complex repair process involving bone resorption and formation. The earliest pathological feature of osteonecrosis is the necrosis of hematopoietic cells and adipocytes, followed by interstitial bone marrow edema (Shah et al., 2015). Vascular injury, inflammation, mechanical stress and increased intraosseous pressure are considered to be important causes of osteonecrosis (Elgaz, Bonig & Bader, 2020; Loi et al., 2016). With the rapid development of cell-based therapies, MSCs have been extensively studied as a treatment for osteonecrosis.

More recent work suggests that EVs derived from MSCs can treat the disease. For example, MSC-derived EVs prevented zoledronic acid-induced senescence in stem cells, osteoblasts, and fibroblasts, while reducing levels of the inflammatory cytokines IL-6 and IL-8 as well as matrix MMP 1 and 3 (Watanabe et al., 2020). Furthermore, MSC-derived EVs can prevent senescence of cells involved in wound healing and the spread of chronic inflammation around senescent cells, thus promoting angiogenesis and bone regeneration and preventing bisphosphonate-related osteonecrosis of the jaw (Watanabe et al., 2020). Exosomes secreted by BMSCs show potential against osteonecrosis of the femoral head (ONFH) by affecting mainly ONFH osteogenesis (Fang, Li & Chen, 2019), whereas exosomes derived from human-induced pluripotent stem cell-derived MSCs can protect against ONFH by promoting local angiogenesis and preventing bone loss (Liu et al., 2017). BMSC-derived EVs carrying miR-148a-3p were found to improve ONFH by suppressing Smad ubiquitination regulatory factor-1, which in turn increased BMSC osteogenic proliferation and differentiation (Huang et al., 2020). Similarly, exosomal miR-135b alleviated ONFH by reducing programmed cell death protein 4(PDCD4)-induced apoptosis of osteoblasts (Zhang et al., 2020b).

Taken together, the available evidence suggests that MSC-derived EVs loaded with miRNAs can alleviate osteonecrosis progression by promoting the proliferation and differentiation of osteoblasts, while enhancing osteogenesis and angiogenesis and reducing inflammatory responses.

**Figure 1:**
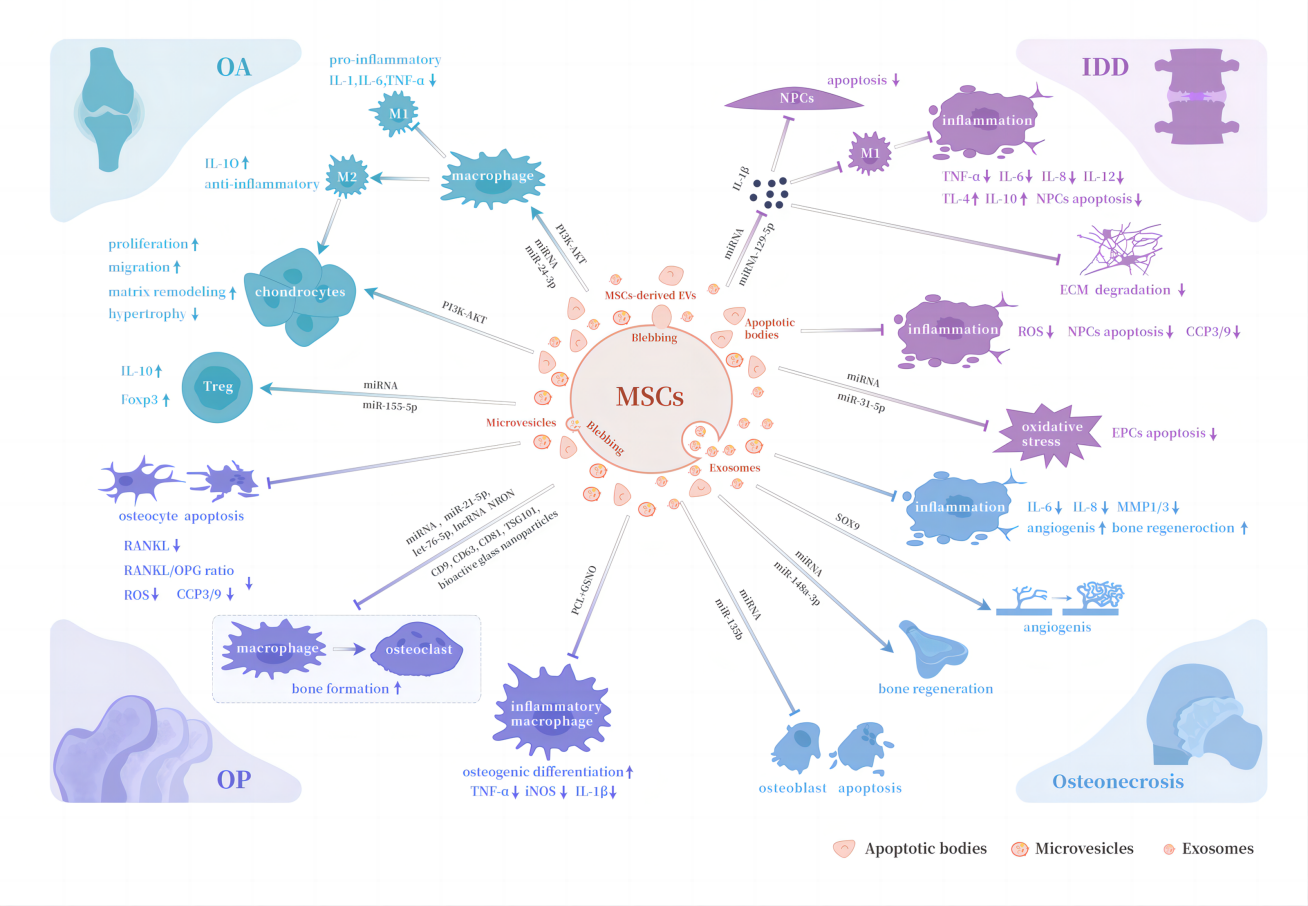
Biological properties of MSCs-derived EVs in orthopedic diseases. Abbreviations: OA, osteoarthritis; OP, osteoporosis; IDD, intervertebral disc degeneration; M1, pro-inflammatory phenotype macrophages; M2, anti-inflammatory phenotype macrophages; Treg , regulatory T cell; RANKL, receptor activator for nuclear factor *κ* B ligand; OPG, osteoprotegerin; NPCs, nucleus pulposus cells; EPCs, endplate chondrocytes; ECM, extracellular matrix; MMP1/3, matrix metalloproteases 1 and 3; iNOS, inducible nitric oxide synthase; PCL, polycaprolactone; GSNO, S-nitrosoglutathione; TNF-α, tumor necrosis factor-α.

Table 1 and Fig. 1 shows and depicts the immunomodulatory functions of different types of MSCs-derived EVs in aforementioned orthopedic diseases.

**Table 1:** Immunomodulatory functions of MSCs-derived EVs in orthopedic diseases.

Diseases	Sources	Cargos	Immunomodulatory functions	Results
Osteoarthritis	BMSCs	Not mentioned	Anti-inflammation; macrophages phenotype regulation	Reduce osteophyte formation and synovial macrophage infiltration; promote chondrocytes proliferation and migration
	hUCMSCs	miR-100-5p	Anti-inflammation; macrophages phenotype regulation	Stimulate chondrocytes activity and matrix remodeling
	ASCs	miR-155-5p; miR-24-3p	Anti-inflammation; macrophages phenotype regulation; Treg proliferation regulation	Promote chondrocytes proliferation and migration
Osteoporosis	BMSCs	lncRNA NRON; bioactive glass nanoparticles	Inhibit osteoclast differentiation of macrophages	Prevent bone loss
	hUCMSCs	CD9; CD63; CD81; TSG101	Inhibit osteoclast differentiation of macrophages	Reduce bone marrow fat accumulation; prevent bone loss
	ASCs	miR-21-5p; let-7b-5p	Anti-inflammation; inhibit oxidative stress; inhibit osteoclast differentiation of macrophages	Inhibit apoptosis of osteocyte
	MSCs	PCL; GSNO	Anti-inflammation	Promote osteogenic differentiation
IDD	MSCs	miR-142-3p; miR-129-5p miR-31-5p	Anti-inflammation; macrophages phenotype regulation; inhibit oxidative stress	Inhibit apoptosis of NPCs and EPCs; inhibit ECM degradation
Osteonecrosis	BMSCs	miR-148a-3p; SOX9 protein	Anti-inflammation	Promote osteogenic differentiation
	MSCs	miR-135b	Anti-inflammation	Reduce apoptosis of osteoblasts; prevent bone loss; induce angiogenesis and bone regeneration

**Notes.**

Abbreviations IDD Intervertebral disc degeneration BMSCs Bone marrow mesenchymal stem cells hUCMSCs human umbilical cord mesenchymal stem cells ASCs adipose mesenchymal stem cells PCL polycaprolactone GSNO S-nitrosoglutathione NPCs nucleus pulposus cells EPCs endplate chondrocytes ECM extracellular matrix

## Conclusions and Expectations

MSC-derived EVs play an important role in bone regeneration, and multiple studies have shown that they can alleviate the progression of orthopedic diseases by protecting chondrocytes from apoptosis; regulating the proliferation, migration, and differentiation of chondrocytes, osteoblasts, and osteoclasts; inhibiting the inflammatory response; regulating osteoimmunity; and promoting angiogenesis. In addition to the succession of biological function of MSCs, MSCs-derived EVs has prominent superiority of lower cytotoxicity and immunogenicity, more convenient manufacturing and storage, and greater stability and bio-activity. These results provide new insights into the pathophysiology of orthopedic diseases, as well as guide the discovery of promising treatments. However, the pathophysiological mechanism of interactions among MSCs-derived EVs, immune system, and skeletal system involving orthopedic diseases still remains inadequate. The lack of clinical evidence of MSCs-derived EVs in the treatment of orthopedic diseases also creates a gap between theory and clinical practice. Future studies should continue with the exploration of the potential and mechanisms of MSCs-derived EVs against orthopedic diseases. At the same time, relevant clinical studies are expected to be furthered with a view to facilitating the transformation from theoretical research to clinical application as soon as possible.

## Additional Information and Declarations

### Data Availability

The following information was supplied regarding data availability: This is a literature review and does not involve raw data or code.

## References

[ref-1] Armas LA, Recker RR (2012). Pathophysiology of osteoporosis: new mechanistic insights. Endocrinology and Metabolism Clinics of North America.

[ref-2] Barile L, Lionetti V, Cervio E, Matteucci M, Gherghiceanu M, Popescu LM, Torre T, Siclari F, Moccetti T, Vassalli G (2014). Extracellular vesicles from human cardiac progenitor cells inhibit cardiomyocyte apoptosis and improve cardiac function after myocardial infarction. Cardiovascular Research.

[ref-3] Bartholomew A, Sturgeon C, Siatskas M, Ferrer K, McIntosh K, Patil S, Hardy W, Devine S, Ucker D, Deans R, Moseley A, Hoffman R (2002). Mesenchymal stem cells suppress lymphocyte proliferation in vitro and prolong skin graft survival in vivo. Experimental Hematology.

[ref-4] Bergsmedh A, Szeles A, Henriksson M, Bratt A, Folkman MJ, Spetz AL, Holmgren L (2001). Horizontal transfer of oncogenes by uptake of apoptotic bodies. Proceedings of the National Academy of Sciences of the United States of America.

[ref-5] Birmingham E, Niebur GL, McHugh PE, Shaw G, Barry FP, McNamara LM (2012). Osteogenic differentiation of mesenchymal stem cells is regulated by osteocyte and osteoblast cells in a simplified bone niche. European Cells & Materials.

[ref-6] Brandau S, Jakob M, Hemeda H, Bruderek K, Janeschik S, Bootz F, Lang S (2010). Tissue-resident mesenchymal stem cells attract peripheral blood neutrophils and enhance their inflammatory activity in response to microbial challenge. Journal of Leukocyte Biology.

[ref-7] Carreras-Planella L, Monguió-Tortajada M, Borràs FE, Franquesa M (2019). Immunomodulatory effect of MSC on B cells is independent of secreted extracellular vesicles. Frontiers in Immunology.

[ref-8] Castanheira FVS, Kubes P (2019). Neutrophils and NETs in modulating acute and chronic inflammation. Blood.

[ref-9] Chen L, Tredget EE, Wu PY, Wu Y (2008). Paracrine factors of mesenchymal stem cells recruit macrophages and endothelial lineage cells and enhance wound healing. PLOS ONE.

[ref-10] Cho DI, Kim MR, Jeong HY, Jeong HC, Jeong MH, Yoon SH, Kim YS, Ahn Y (2014). Mesenchymal stem cells reciprocally regulate the M1/M2 balance in mouse bone marrow-derived macrophages. Experimental & Molecular Medicine.

[ref-11] Clynes MA, Harvey NC, Curtis EM, Fuggle NR, Dennison EM, Cooper C (2020). The epidemiology of osteoporosis. British Medical Bulletin.

[ref-12] Colucci S, Brunetti G, Rizzi R, Zonno A, Mori G, Colaianni G, Del Prete D, Faccio R, Liso A, Capalbo S, Liso V, Zallone A, Grano M (2004). T cells support osteoclastogenesis in an in vitro model derived from human multiple myeloma bone disease: the role of the OPG/TRAIL interaction. Blood.

[ref-13] Corcione A, Benvenuto F, Ferretti E, Giunti D, Cappiello V, Cazzanti F, Risso M, Gualandi F, Mancardi GL, Pistoia V, Uccelli A (2006). Human mesenchymal stem cells modulate B-cell functions. Blood.

[ref-14] Cui S, Zhang L (2021). microRNA-129-5p shuttled by mesenchymal stem cell-derived extracellular vesicles alleviates intervertebral disc degeneration via blockade of LRG1-mediated p38 MAPK activation. Journal of Tissue Engineering.

[ref-15] Dar HY, Azam Z, Anupam R, Mondal RK, Srivastava RK (2018). Osteoimmunology: the Nexus between bone and immune system. Frontiers in Bioscience.

[ref-16] Dimitriou R, Jones E, McGonagle D, Giannoudis PV (2011). Bone regeneration: current concepts and future directions. BMC Medicine.

[ref-17] Ding DC, Shyu WC, Lin SZ (2011). Mesenchymal stem cells. Cell Transplantation.

[ref-18] Ding F, Shao ZW, Xiong LM (2013). Cell death in intervertebral disc degeneration. Apoptosis.

[ref-19] Dou C, Ding N, Zhao C, Hou T, Kang F, Cao Z, Liu C, Bai Y, Dai Q, Ma Q, Luo F, Xu J, Dong S (2018). Estrogen deficiency-mediated M2 macrophage osteoclastogenesis contributes to M1/M2 ratio alteration in ovariectomized osteoporotic mice. Journal of Bone and Mineral Research.

[ref-20] Dowdell J, Erwin M, Choma T, Vaccaro A, Iatridis J, Cho SK (2017). Intervertebral disk degeneration and repair. Neurosurgery.

[ref-21] Ela S, Mäger I, Breakefield XO, Wood MJ (2013). Extracellular vesicles: biology and emerging therapeutic opportunities. Nature Reviews Drug Discovery.

[ref-22] Elgaz S, Bonig H, Bader P (2020). Mesenchymal stromal cells for osteonecrosis. Journal of Translational Medicine.

[ref-23] Ensrud KE, Crandall CJ (2019). Bisphosphonates for postmenopausal osteoporosis. Jama.

[ref-24] Fang S, Li Y, Chen P (2019). Osteogenic effect of bone marrow mesenchymal stem cell-derived exosomes on steroid-induced osteonecrosis of the femoral head. Drug Design, Development and Therapy.

[ref-25] Fierro FA, Nolta JA, Adamopoulos IE (2017). Concise review: stem cells in osteoimmunology. Stem Cells.

[ref-26] Freemont AJ, Watkins A, Le Maitre C, Jeziorska M, Hoyland JA (2002). Current understanding of cellular and molecular events in intervertebral disc degeneration: implications for therapy. Journal of Pathology.

[ref-27] Gatti S, Bruno S, Deregibus MC, Sordi A, Cantaluppi V, Tetta C, Camussi G (2011). Microvesicles derived from human adult mesenchymal stem cells protect against ischaemia-reperfusion-induced acute and chronic kidney injury. Nephrology, Dialysis, Transplantation.

[ref-28] Glennie S, Soeiro I, Dyson PJ, Lam EW, Dazzi F (2005). Bone marrow mesenchymal stem cells induce division arrest anergy of activated T cells. Blood.

[ref-29] Goodman SB, Maruyama M (2020). Inflammation, bone healing and osteonecrosis: from bedside to bench. Journal of Inflammation Research.

[ref-30] Griffin MD, Ryan AE, Alagesan S, Lohan P, Treacy O, Ritter T (2013). Anti-donor immune responses elicited by allogeneic mesenchymal stem cells: what have we learned so far?. Immunology and Cell Biology.

[ref-31] Griffin TM, Scanzello CR (2019). Innate inflammation and synovial macrophages in osteoarthritis pathophysiology. Clinical and Experimental Rheumatology 37 Suppl.

[ref-32] Hernigou P, Trousselier M, Roubineau F, Bouthors C, Chevallier N, Rouard H, Flouzat-Lachaniette CH (2016). Stem cell therapy for the treatment of hip osteonecrosis: a 30-year review of progress. Clinics in Orthopedic Surgery.

[ref-33] Hessvik NP, Llorente A (2018). Current knowledge on exosome biogenesis and release. Cellular and Molecular Life Science.

[ref-34] Horton JE, Raisz LG, Simmons HA, Oppenheim JJ, Mergenhagen SE (1972). Bone resorbing activity in supernatant fluid from cultured human peripheral blood leukocytes. Science.

[ref-35] Hu S, Xing H, Zhang J, Zhu Z, Yin Y, Zhang N, Qi Y (2022). Mesenchymal stem cell-derived extracellular vesicles: immunomodulatory effects and potential applications in intervertebral disc degeneration. Stem Cells International.

[ref-36] Hu Y, Zhang Y, Ni CY, Chen CY, Rao SS, Yin H, Huang J, Tan YJ, Wang ZX, Cao J, Liu ZZ, Xie PL, Wu B, Luo J, Xie H (2020). Human umbilical cord mesenchymal stromal cells-derived extracellular vesicles exert potent bone protective effects by CLEC11A-mediated regulation of bone metabolism. Theranostics.

[ref-37] Huang S, Li Y, Wu P, Xiao Y, Duan N, Quan J, Du W (2020). microRNA-148a-3p in extracellular vesicles derived from bone marrow mesenchymal stem cells suppresses SMURF1 to prevent osteonecrosis of femoral head. Journal of Cellular and Molecular Medicine.

[ref-38] Hunter DJ, March L, Chew M (2020). Osteoarthritis in 2020 and beyond: a lancet commission. Lancet.

[ref-39] Ibrahim SA, Khan YS (2022). Histology, extracellular vesicles. StatPearls.

[ref-40] Jamalpoor Z, Asgari A, Lashkari MH, Mirshafiey A, Mohsenzadegan M (2018). Modulation of macrophage polarization for bone tissue engineering applications. Iranian Journal of Allergy, Asthma and Immunology.

[ref-41] Jiang D, Muschhammer J, Qi Y, Kügler A, De Vries JC, Saffarzadeh M, Sindrilaru A, Beken SV, Wlaschek M, Kluth MA, Ganss C, Frank NY, Frank MH, Preissner KT, Scharffetter-Kochanek K (2016). Suppression of neutrophil-mediated tissue damage-a novel skill of mesenchymal stem cells. Stem Cells.

[ref-42] Keshtkar S, Azarpira N, Ghahremani MH (2018). Mesenchymal stem cell-derived extracellular vesicles: novel frontiers in regenerative medicine. Stem Cell Research & Therapy.

[ref-43] Kim GB, Shon OJ, Seo MS, Choi Y, Park WT, Lee GW (2021). Mesenchymal stem cell-derived exosomes and their therapeutic potential for osteoarthritis. Biology.

[ref-44] Kordelas L, Rebmann V, Ludwig AK, Radtke S, Ruesing J, Doeppner TR, Epple M, Horn PA, Beelen DW, Giebel B (2014). MSC-derived exosomes: a novel tool to treat therapy-refractory graft-versus-host disease. Leukemia.

[ref-45] Kovach TK, Dighe AS, Lobo PI, Cui Q (2015). Interactions between MSCs and immune cells: implications for bone healing. Journal of Immunology Research.

[ref-46] Lai FW, Lichty BD, Bowdish DM (2015). Microvesicles: ubiquitous contributors to infection and immunity. Journal of Leukocyte Biology.

[ref-47] Lai J, Huang C, Guo Y, Rao L (2022). Engineered extracellular vesicles and their mimics in cardiovascular diseases. Journal of Controlled Release.

[ref-48] Lee J, Byun H, Madhurakkat Perikamana SK, Lee S, Shin H (2019). Current advances in immunomodulatory biomaterials for bone regeneration. Advanced Healthcare Materials.

[ref-49] Lee KS, Lee J, Kim HK, Yeom SH, Woo CH, Jung YJ, Yun YE, Park SY, Han J, Kim E, Sul JH, Jung JM, Park JH, Choi JS, Cho YW, Jo DG (2021). Extracellular vesicles from adipose tissue-derived stem cells alleviate osteoporosis through osteoprotegerin and miR-21-5p. Journal of Extracellular Vesicles.

[ref-50] Li K, Yan G, Huang H, Zheng M, Ma K, Cui X, Lu D, Zheng L, Zhu B, Cheng J, Zhao J (2022). Anti-inflammatory and immunomodulatory effects of the extracellular vesicles derived from human umbilical cord mesenchymal stem cells on osteoarthritis via M2 macrophages. Journal of Nanobiotechnology.

[ref-51] Li S, Stöckl S, Lukas C, Götz J, Herrmann M, Federlin M, Grässel S (2020). hBMSC-derived extracellular vesicles attenuate IL-1 *β*-Induced catabolic effects on OA-Chondrocytes by regulating pro-inflammatory signaling pathways. Frontiers in Bioengineering and Biotechnology.

[ref-52] Liu X, Li Q, Niu X, Hu B, Chen S, Song W, Ding J, Zhang C, Wang Y (2017). Exosomes secreted from human-induced pluripotent stem cell-derived mesenchymal stem cells prevent osteonecrosis of the femoral head by promoting angiogenesis. International Journal of Biological Sciences.

[ref-53] Livshits G, Kalinkovich A (2022). Targeting chronic inflammation as a potential adjuvant therapy for osteoporosis. Life Sciences.

[ref-54] Loi F, Córdova LA, Pajarinen J, Lin TH, Yao Z, Goodman SB (2016). Inflammation, fracture and bone repair. Bone.

[ref-55] Lu L, Xu A, Gao F, Tian C, Wang H, Zhang J, Xie Y, Liu P, Liu S, Yang C, Ye Z, Wu X (2021). Mesenchymal stem cell-derived exosomes as a novel strategy for the treatment of intervertebral disc degeneration. Frontiers in Cell and Developmental Biology.

[ref-56] Luo Z, Lin J, Sun Y, Wang C, Chen J (2021). Bone marrow stromal cell-derived exosomes promote muscle healing following contusion through macrophage polarization. Stem Cells and Development.

[ref-57] Ma M, Tan Z, Li W, Zhang H, Liu Y, Yue C (2022). Osteoimmunology and osteonecrosis of the femoral head. Bone & Joint Research.

[ref-58] Mendt M, Rezvani K, Shpall E (2019). Mesenchymal stem cell-derived exosomes for clinical use. Bone Marrow Transplantation.

[ref-59] Meng Q, Qiu B (2020). Exosomal MicroRNA-320a derived from mesenchymal stem cells regulates rheumatoid arthritis fibroblast-like synoviocyte activation by suppressing CXCL9 expression. Frontiers in Physiology.

[ref-60] Mianehsaz E, Mirzaei HR, Mahjoubin-Tehran M, Rezaee A, Sahebnasagh R, Pourhanifeh MH, Mirzaei H, Hamblin MR (2019). Mesenchymal stem cell-derived exosomes: a new therapeutic approach to osteoarthritis?. Stem Cell Research & Therapy.

[ref-61] Nicola MDi, Carlo-Stella C, Magni M, Milanesi M, Longoni PD, Matteucci P, Grisanti S, Gianni AM (2002). Human bone marrow stromal cells suppress T-lymphocyte proliferation induced by cellular or nonspecific mitogenic stimuli. Blood.

[ref-62] Ono T, Takayanagi H (2017). Osteoimmunology in bone fracture healing. Current Osteoporosis Reports.

[ref-63] Park BH, Jeong ES, Lee S, Jang JH (2021). Bio-functionalization and in-vitro evaluation of titanium surface with recombinant fibronectin and elastin fragment in human mesenchymal stem cell. PLOS ONE.

[ref-64] Phinney DG, Pittenger MF (2017). Concise review: MSC-derived exosomes for cell-free therapy. Stem Cells.

[ref-65] Pittenger MF, Mackay AM, Beck SC, Jaiswal RK, Douglas R, Mosca JD, Moorman MA, Simonetti DW, Craig S, Marshak DR (1999). Multilineage potential of adult human mesenchymal stem cells. Science.

[ref-66] Ragni E, Colombini A, Viganò M, Libonati F, Perucca Orfei C, Zagra L, De Girolamo L (2021). Cartilage protective and immunomodulatory features of osteoarthritis synovial fluid-treated adipose-derived mesenchymal stem cells secreted factors and extracellular vesicles-embedded miRNAs. Cell.

[ref-67] Ratajczak MZ, Ratajczak J (2020). Extracellular microvesicles/exosomes: discovery, disbelief, acceptance, and the future?. Leukemia.

[ref-68] Ren L, Song ZJ, Cai QW, Chen RX, Zou Y, Fu Q, Ma YY (2019). Adipose mesenchymal stem cell-derived exosomes ameliorate hypoxia/serum deprivation-induced osteocyte apoptosis and osteocyte-mediated osteoclastogenesis in vitro. Biochemical and Biophysical Research Communications.

[ref-69] Sacitharan PK (2019). Ageing and osteoarthritis. Subcellular Biochemistry.

[ref-70] Saxena Y, Routh S, Mukhopadhaya A (2021). Immunoporosis: role of innate immune cells in osteoporosis. Frontiers in Immunology.

[ref-71] Shah KN, Racine J, Jones LC, Aaron RK (2015). Pathophysiology and risk factors for osteonecrosis. Current Reviews in Musculoskeletal Medicine.

[ref-72] Shahrezaee M, Salehi M, Keshtkari S, Oryan A, Kamali A, Shekarchi B (2018). In vitro and in vivo investigation of PLA/PCL scaffold coated with metformin-loaded gelatin nanocarriers in regeneration of critical-sized bone defects. Nanomedicine.

[ref-73] Sokolove J, Lepus CM (2013). Role of inflammation in the pathogenesis of osteoarthritis: latest findings and interpretations. Therapeutic Advances in Musculoskeletal Disease.

[ref-74] Taghiyar L, Jahangir S, Khozaei Ravari M, Shamekhi MA, Eslaminejad MB (2021). Cartilage repair by mesenchymal stem cell-derived exosomes: preclinical and clinical trial update and perspectives. Advances in Experimental Medicine and Biology.

[ref-75] Takayanagi H, Ogasawara K, Hida S, Chiba T, Murata S, Sato K, Takaoka A, Yokochi T, Oda H, Tanaka K, Nakamura K, Taniguchi T (2000). T-cell-mediated regulation of osteoclastogenesis by signalling cross-talk between RANKL and IFN-gamma. Nature.

[ref-76] Tang S, Chen P, Zhang H, Weng H, Fang Z, Chen C, Peng G, Gao H, Hu K, Chen J, Chen L, Chen X (2021). Comparison of curative effect of human umbilical cord-derived mesenchymal stem cells and their small extracellular vesicles in treating osteoarthritis. International Journal of Nanomedicine.

[ref-77] Tian X, Wei W, Cao Y, Ao T, Huang F, Javed R, Wang X, Fan J, Zhang Y, Liu Y, Lai L, Ao Q (2022). Gingival mesenchymal stem cell-derived exosomes are immunosuppressive in preventing collagen-induced arthritis. Journal of Cellular and Molecular Medicine.

[ref-78] Tjempakasari A, Suroto H, Santoso D (2021). Mesenchymal stem cell senescence and osteogenesis. Medicina.

[ref-79] Todorova D, Simoncini S, Lacroix R, Sabatier F, Dignat-George F (2017). Extracellular vesicles in angiogenesis. Circulation Research.

[ref-80] Tsiapalis D, O’Driscoll L (2020). Mesenchymal stem cell derived extracellular vesicles for tissue engineering and regenerative medicine applications. Cell.

[ref-81] van der Pol E, Böing AN, Harrison P, Sturk A, Nieuwland R (2012). Classification, functions, and clinical relevance of extracellular vesicles. Pharmacological Reviews.

[ref-82] Vig S, Fernandes MH (2022). Bone cell exosomes and emerging strategies in bone engineering. Biomedicines.

[ref-83] Wang X, Ao J, Lu H, Zhao Q, Ma Y, Zhang J, Ren H, Zhang Y (2020). Osteoimmune modulation and guided osteogenesis promoted by barrier membranes incorporated with S-Nitrosoglutathione (GSNO) and mesenchymal stem cell-derived exosomes. International Journal of Nanomedicine.

[ref-84] Watanabe J, Sakai K, Urata Y, Toyama N, Nakamichi E, Hibi H (2020). Extracellular vesicles of stem cells to prevent BRONJ. Journal of Dental Research.

[ref-85] Wei F, Li Z, Crawford R, Xiao Y, Zhou Y (2019). Immunoregulatory role of exosomes derived from differentiating mesenchymal stromal cells on inflammation and osteogenesis. Journal of Tissue Engineering and Regenerative Medicine.

[ref-86] Woo CH, Kim HK, Jung GY, Jung YJ, Lee KS, Yun YE, Han J, Lee J, Kim WS, Choi JS, Yang S, Park JH, Jo DG, Cho YW (2020). Small extracellular vesicles from human adipose-derived stem cells attenuate cartilage degeneration. Journal of Extracellular Vesicles.

[ref-87] Woodell-May JE, Sommerfeld SD (2020). Role of inflammation and the immune system in the progression of osteoarthritis. Journal of Orthopaedic Research.

[ref-88] Xia C, Zeng Z, Fang B, Tao M, Gu C, Zheng L, Wang Y, Shi Y, Fang C, Mei S, Chen Q, Zhao J, Lin X, Fan S, Jin Y, Chen P (2019). Mesenchymal stem cell-derived exosomes ameliorate intervertebral disc degeneration via anti-oxidant and anti-inflammatory effects. Free Radical Biology and Medicine.

[ref-89] Xie L, Chen Z, Liu M, Huang W, Zou F, Ma X, Tao J, Guo J, Xia X, Lyu F, Wang H, Zheng C, Jiang J (2020). MSC-derived exosomes protect vertebral endplate chondrocytes against apoptosis and calcification via the miR-31-5p/ATF6 axis. Molecular Therapy - Nucleic Acids.

[ref-90] Yang Z, Liu X, Zhao F, Yao M, Lin Z, Yang Z, Liu C, Liu Y, Chen X, Du C (2022). Bioactive glass nanoparticles inhibit osteoclast differentiation and osteoporotic bone loss by activating lncRNA NRON expression in the extracellular vesicles derived from bone marrow mesenchymal stem cells. Biomaterials.

[ref-91] Yao Y, Cai X, Ren F, Ye Y, Wang F, Zheng C, Qian Y, Zhang M (2021). The macrophage-osteoclast axis in osteoimmunity and osteo-related diseases. Frontiers in Immunology.

[ref-92] Zhang J, Rong Y, Luo C, Cui W (2020a). Bone marrow mesenchymal stem cell-derived exosomes prevent osteoarthritis by regulating synovial macrophage polarization. Aging.

[ref-93] Zhang QZ, Su WR, Shi SH, Wilder-Smith P, Xiang AP, Wong A, Nguyen AL, Kwon CW, Le AD (2010). Human gingiva-derived mesenchymal stem cells elicit polarization of m2 macrophages and enhance cutaneous wound healing. Stem Cells.

[ref-94] Zhang W, Ge W, Li C, You S, Liao L, Han Q, Deng W, Zhao RC (2004). Effects of mesenchymal stem cells on differentiation, maturation, and function of human monocyte-derived dendritic cells. Stem Cells and Development.

[ref-95] Zhang X, You JM, Dong XJ, Wu Y (2020b). Administration of mircoRNA-135b-reinforced exosomes derived from MSCs ameliorates glucocorticoid-induced osteonecrosis of femoral head (ONFH) in rats. Journal of Cellular and Molecular Medicine.

[ref-96] Zhao AG, Shah K, Cromer B, Sumer H (2020a). Mesenchymal stem cell-derived extracellular vesicles and their therapeutic potential. Stem Cells International.

[ref-97] Zhao X, Zhao Y, Sun X, Xing Y, Wang X, Yang Q (2020b). Immunomodulation of MSCs and MSC-derived extracellular vesicles in osteoarthritis. Frontiers in Bioengineering and Biotechnology.

[ref-98] Zhou F, Zhang G, Wu Y, Xiong Y (2022). Inflammasome complexes: crucial mediators in osteoimmunology and bone diseases. International Immunopharmacology.

[ref-99] Zhu L, Shi Y, Liu L, Wang H, Shen P, Yang H (2020). Mesenchymal stem cells-derived exosomes ameliorate nucleus pulposus cells apoptosis via delivering miR-142-3p: therapeutic potential for intervertebral disc degenerative diseases. Cell Cycle.

